# ﻿Three new species of *Pseudopoda* Jäger, 2000 (Araneae, Sparassidae, Heteropodinae) from Qizimeishan National Nature Reserve of Hubei, China

**DOI:** 10.3897/zookeys.1214.130101

**Published:** 2024-10-03

**Authors:** Jian Chang, He Zhang, Jie Liu, Yang Zhu, Changyong Liu, Kuai Chen, Changhao Hu

**Affiliations:** 1 The State Key Laboratory of Biocatalysis and Enzyme Engineering of China, College of Life Science, Hubei University, Wuhan 430062, Hubei, China; 2 Hubei Key Laboratory of Regional Development and Environmental Response, Faculty of Resources and Environmental Science, Hubei University, Wuhan 430062, Hubei, China; 3 Guo Shoujing Innovation College, Xingtai University, Xingtai 054001, Hebei, China; 4 Qizimeishan National Nature Reserve Administration, Xuan’en 445500, Hubei, China; 5 Hubei Broad Nature Technology Service Co., Ltd., Wuhan 430079, Hubei, China

**Keywords:** Biodiversity, high-altitude niche, huntsman spiders, morphology, taxonomy, Yunnan-Guizhou Plateau

## Abstract

The Qizimeishan National Nature Reserve is situated in the southwestern region of Hubei Province, adjacent to the northeastern edge of the Yunnan-Guizhou Plateau. A survey of spiders of this reserve was conducted recently, leading to the discovery of three new species of the genus *Pseudopoda* Jäger, 2000: *P.arcuata* Zhang, J. Liu & Hu, **sp. nov.** (♀), *P.qizimeishanensis* Zhang, J. Liu & Hu, **sp. nov.** (♂, ♀) and *P.weimiani* Zhang, J. Liu & Hu, **sp. nov.** (♂, ♀). Diagnoses, descriptions, photos, and a distribution map are provided.

## ﻿Introduction

The Qizimeishan National Nature Reserve is located in Xuan’en County, southwest Hubei, with a total area of 345.5 km^2^ and the highest peak about 2010 m above sea level (Liu et al. 2006). It mainly protects the middle subtropical mountain evergreen broad-leaved forest and the subalpine sphagnum marshes wetland areas. The reserve is rich in wildlife resources and has been listed as a key area of biodiversity in the country by the China Biodiversity Conservation Action Plan (Xu et al. 2006).

The genus *Pseudopoda* Jäger, 2000 is the largest genus of the family Sparassidae Bertkau, 1872, with 256 known species (World Spider Catalog 2024). Currently, 155 *Pseudopoda* species are known in China and five in Hubei (Quan et al. 2014; Zhang et al. 2023; Gong et al. 2023; Wen et al. 2024). Recently, a series of taxonomic works on the genus was published: Wen et al. (2024) described one new species from Hubei; Wu et al. (2024) described three new species from China, Laos, and Thailand; Gong et al. (2023) described a new species from Hubei; Deng et al. (2023) described four new species from Chongqing; and Zhang et al. (2023) described 99 new species from East, South and Southeast Asia. *Pseudopoda* species are small to large spiders, living primarily in the leaf litter and less commonly on vegetation (Jäger et al. 2015).

A survey of spiders in the Qizimeishan National Nature Reserve carried out by colleagues of the Hubei University from June to July 2023 yielded three new species of *Pseudopoda*, which are described herein.

## ﻿Material and methods

Specimens were examined using an Olympus SZX7 stereo microscope. Photographs were taken with a Leica M205C stereo microscope, and final multifocal images were produced with Helicon Focus (version 7.7.0). The male palps were examined and photographed after dissection. The epigynes were examined after being dissected from the spider’s body. All morphological measurements were calculated using a Leica M205C stereo microscope. Eye diameters were taken at the widest point. Legs and palps measurements were given as total length (femur, patella, tibia, metatarsus [absent in palp], tarsus). The terminologies used in text and figure legends follow Quan et al. (2014). Spination follows that given in Davies (1994). All measurements were in millimetres (mm). The specimens examined in this study were deposited in the Centre for Behavioural Ecology and Evolution (CBEE), College of Life Sciences, Hubei University in Wuhan.

Abbreviations in text and figures: **AB**, anterior bands; **ALE**, anterior lateral eyes; **AME**, anterior median eyes; **C**, conductor; **CH**, clypeus height; **CO**, copulatory opening; **dRTA**, dorsal retrolateral tibial apophysis; **DS**, dorsal shield of prosoma; **E**, embolus; **EF**, epigynal field; **EP**, embolic projection; **FD**, fertilization duct; **Fe**, femur; **FW**, first winding; **LL**, lateral lobes; **Mt**, metatarsus; **OS**, opisthosoma; **Pa**, patella; **PLE**, posterior lateral eyes; **PME**, posterior median eyes; **Pp**, palp; **RTA**, retrolateral tibial apophysis; **S**, spermathecae; **Sp**, spermophor; **ST**, subtegulum; **T**, tegulum; **Ti**, tibia; **vRTA**, ventral retrolateral tibial apophysis; **I, II, III, IV**, legs I to IV.

## ﻿Result

### ﻿Taxonomy

**Family Sparassidae Bertkau, 1872**

**Subfamily Heteropodinae Thorell, 1873**

#### ﻿Genus Pseudopoda

Taxon classificationAnimaliaAraneaeSparassidae

Jäger, 2000

6CDA4E81-148D-586B-9CBD-EA164F3B65EF

##### Type species.

*Pseudopodaprompta* (O. Pickard-Cambridge, 1885).

##### Diagnosis.

Male *Pseudopoda* species can be diagnosed by the following combination of characters: 1) embolus at least in its basal part broadened and flattened; 2) conductor membranous (absent in some species); and 3) retrolateral tibial apophysis arising proximally or mesially from the tibia. Females can be diagnosed by the following combination of characters: 1) epigyne with lateral lobes extending distinctly beyond epigastric furrow, and covering median septum in most species; 2) first winding membranous, and with bent margins in most species; and 3) first winding or first winding and lateral lobes covering the internal duct system (Jäger, 2000; Jiang et al. 2018; Zhang et al. 2023; Wu et al. 2024).

##### Distribution.

East, South and Southeast Asia.

#### ﻿ Pseudopoda arcuata

Taxon classificationAnimaliaAraneaeSparassidae

Zhang, J. Liu & Hu sp. nov.

4D2D15DF-6FCB-5203-A686-A5BB961C4D10

https://zoobank.org/79C05FD8-0C12-4853-A41E-E336BF9B01DD

Figs 2 , 3 , 10

##### Type material.

***Holotype*** • female: China, Hubei Province: Enshi Tujia and Miao Autonomous Prefecture, Xuan’en County, Qizimeishan National Nature Reserve, Chunmuying Town, Shaiping Village; 29°57'47.52"N, 109°45'20.60"E; elev. 1822 m; 31 July 2023; Changhao Hu & Mian Wei leg. (CBEE, QZMS00602). ***Paratype*** • 1 female, Enshi Tujia and Miao Autonomous Prefecture, Xuan’en County, Qizimeishan National Nature Reserve, Chunmuying Town, Huoshaobao; 30°1'27.03"N, 109°45'23.23"E; elev. 1919 m; 13 July 2023; Changhao Hu & Mian Wei leg. (CBEE, QZMS00582).

##### Etymology.

The specific epithet is a Latin word meaning “arc-shaped”, referring to the arc-shaped LL; adjective.

##### Diagnosis.

The female of *P.arcuata* Zhang, J. Liu & Hu, sp. nov. resembles that of *P.allantoides* Zhang, Jäger & Liu, 2023 (cf. fig. 2A–C vs. fig. 8A–C in Zhang et al. 2023) by having curved anterior and posterior margins of LL, but can be recognised by: 1) S extending horizontally; and 2) FD arising anteriorly from S (vs. S extending longitudinally, FD arising medially from S in *P.allantoides*).

**Female: *Measurements***: Small-sized. Body length 6.0, DS length 3.0, width 2.8; OS length 2.9, width 2.0. ***Eyes***: AME 0.13, ALE 0.19, PME 0.22, PLE 0.23, AME–AME 0.11, AME–ALE 0.07, PME–PME 0.21, PME–PLE 0.28, AME–PME 0.19, ALE–PLE 0.26, CHAME 0.23, CHALE 0.19. ***Spination***: Pp 131, 101, 2121, 1014; Fe I–III 323, IV 321; Pa I–IV 000; Ti I–II 2221, III–IV 2126; Mt I–II 2024, III–IV 2026. ***Measurements of palp and legs***: Pp 3.4 (1.1, 0.8, 0.4, –, 1.1), I 9.3 (2.5, 1.1, 2.6, 2.2, 0.9), II 12.1 (3.2, 1.4, 3.6, 2.8, 1.1), III 7.3 (2.0, 0.7, 2.1, 1.7, 0.8), IV 9.2 (2.9, 0.9, 2.2, 2.3, 0.9). Leg formula: II-I-IV-III. Promargin of chelicerae with three teeth, retromargin with four teeth, cheliceral furrow with c. 28 denticles.

***Epigyne*** (Fig. 2A–C): As in diagnosis. EF 2 times wider than long, without obvious AB. Anterior and posterior margins of LL almost parallel and strongly curved. FW covering the whole S. S with enlarged terminal. FD narrow and long, laterad.

***Colouration*** (Fig. 3A, B): DS yellow, with black marks. Fovea black. Legs with black spots. Dorsal OS brown, with black spots, ventral OS dark yellow, with several black spots, with a brown patch in front of spinnerets.

**Male**: Unknown.

##### Distribution.

China (Hubei Province) (Fig. 10).

#### ﻿ Pseudopoda qizimeishanensis

Taxon classificationAnimaliaAraneaeSparassidae

Zhang, J. Liu & Hu sp. nov.

70F158B3-D851-5EED-AEA3-08964ADD207A

https://zoobank.org/DE4E69FA-D226-4043-A461-1E00BDCCB3B7

**Figure 1.**
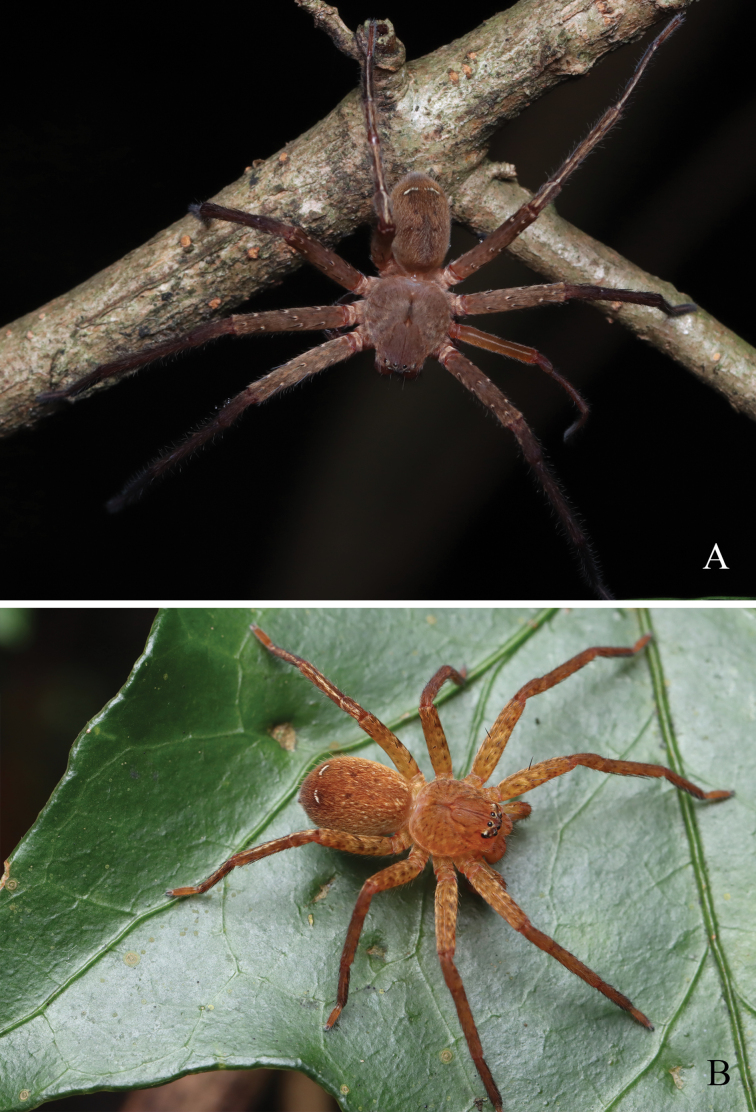
*Pseudopodaqizimeishanensis* Zhang, J. Liu & Hu, sp. nov. (photos by Mian Wei) **A** male **B** female.

Figs 1 , 4 , 5 , 6 , 10

##### Type material.

***Holotype*** • male: China, Hubei Province: Enshi Tujia and Miao Autonomous Prefecture, Xuan’en County, Changtanhe Dong Autonomous Township, Qizimeishan National Nature Reserve, Qizimeishan Mountain; 30°1'45.19"N, 109°43'45.42"E; elev. 1270 m; 6–11 July 2023; Changhao Hu & Mian Wei leg. (CBEE, QZMS00902). ***Paratypes*** • 8 males and 10 females, with same data as for holotype (CBEE, QZMS02441–QZMS02458).

**Figure 2.**
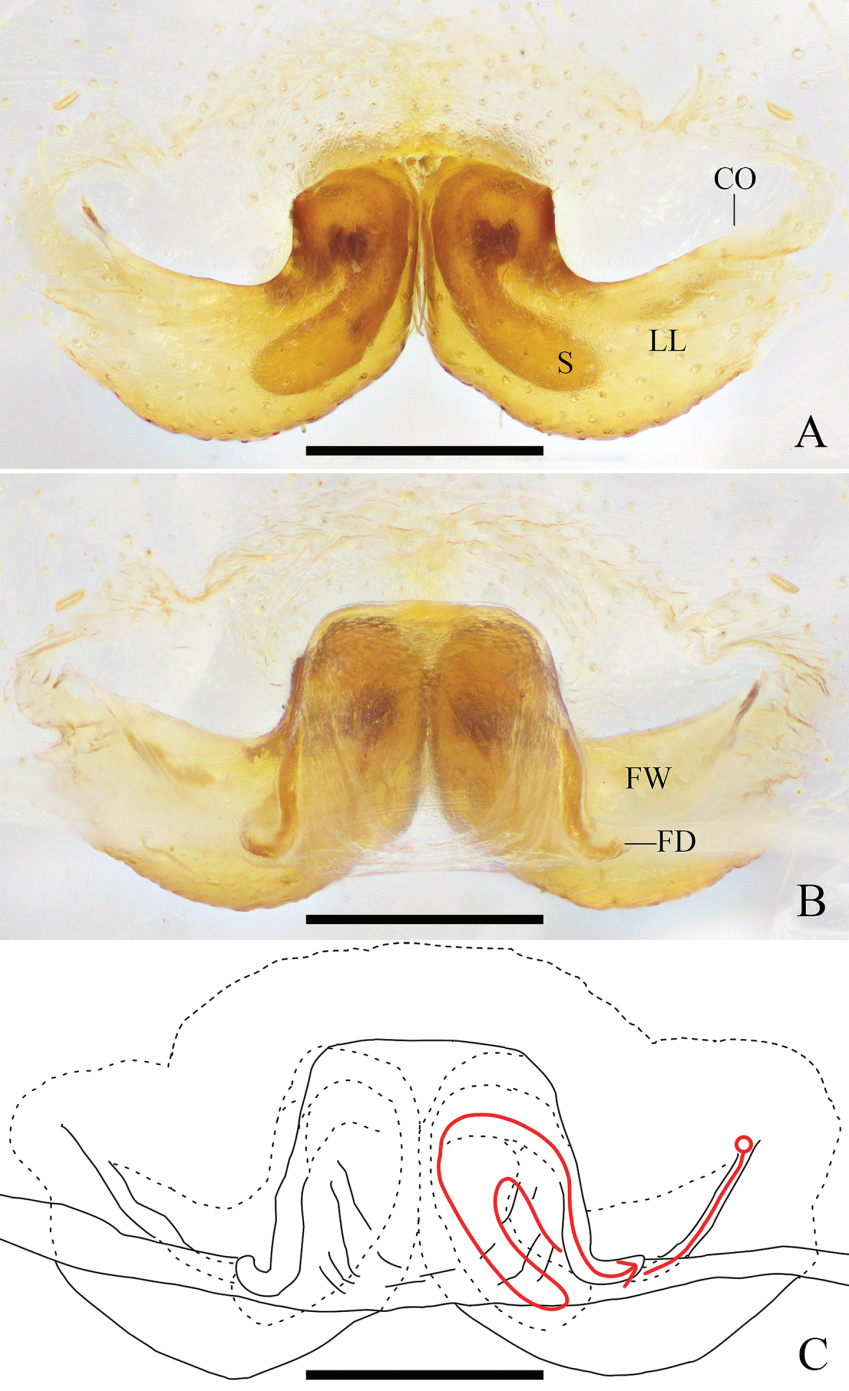
*Pseudopodaarcuata* Zhang, J. Liu & Hu, sp. nov., female **A** epigyne, ventral **B** vulva, dorsal **C** vulva, dorsal; red line represents schematic course of internal duct system. Abbreviations: CO, copulatory opening; FD, fertilization duct; FW, first winding; LL, lateral lobes; S, spermathecae. Scale bars: 0.2 mm.

**Figure 3.**
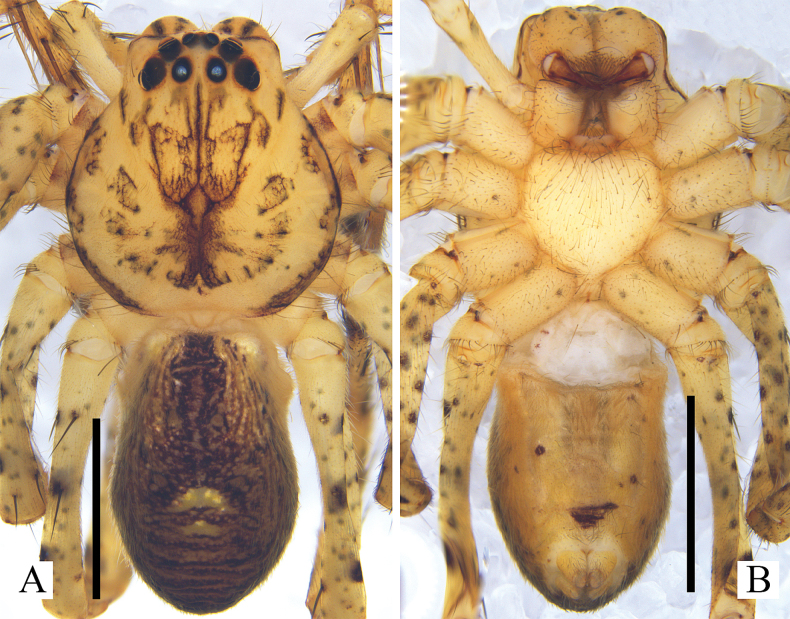
*Pseudopodaarcuata* Zhang, J. Liu & Hu, sp. nov., female habitus (**A** dorsal **B** ventral). Scale bars: 2 mm.

##### Etymology.

The specific epithet is derived from the type locality, the Qizimeishan Mountain; adjective.

##### Diagnosis.

**Figure 4.**
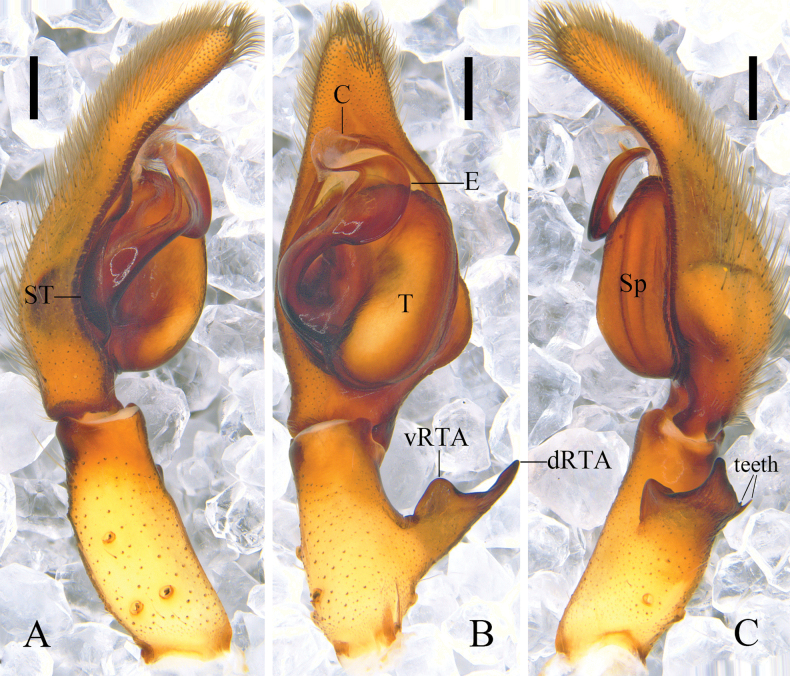
*Pseudopodaqizimeishanensis* Zhang, J. Liu & Hu, sp. nov., left male palp (**A** prolateral **B** ventral **C** retrolateral). Abbreviations: C, conductor; dRTA, dorsal retrolateral tibial apophysis; E, embolus; Sp, spermophor; ST, subtegulum; T, tegulum; vRTA, ventral retrolateral tibial apophysis. Scale bars: 0.5 mm.

**Figure 5.**
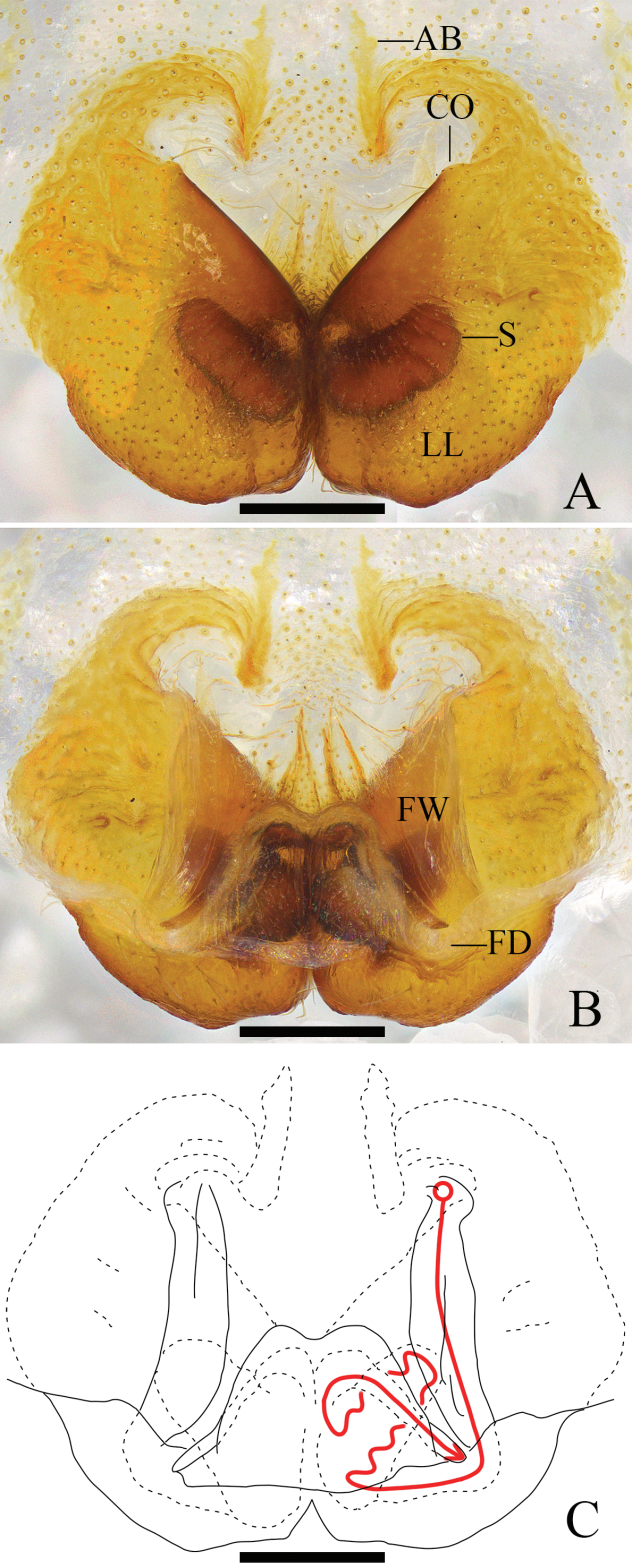
*Pseudopodaqizimeishanensis* Zhang, J. Liu & Hu, sp. nov., female **A** epigyne, ventral **B** vulva, dorsal **C** vulva, dorsal; red line represents schematic course of internal duct system. Abbreviations: AB, anterior bands; CO, copulatory opening; FD, fertilization duct; FW, first winding; LL, lateral lobes; S, spermathecae. Scale bars: 0.5 mm.

The male of *P.qizimeishanensis* Zhang, J. Liu & Hu, sp. nov. resembles that of *P.baoshanensis* Zhang, Jäger & Liu, 2023 (cf. fig. 4A–C vs. fig. 26A–C in Zhang et al. 2023) by having the expanded E, but can be recognised by: 1) RTA long, arising basally from Ti; 2) T without prolaterad outgrowth; and 3) tip of E pointing 11 o’clock (vs. RTA short, arising medially from Ti, T with prolaterad outgrowth, tip of E pointing 7 o’clock in *P.baoshanensis*). The female of *P.qizimeishanensis* Zhang, J. Liu & Hu, sp. nov. resembles that of *P.nanyueensis* Tang & Yin, 2000 (cf. fig. 5A–C vs. figs 2, 3 in Tang and Yin 2000) by: 1) anterior margins of LL V-shaped; 2) anterior margins of LL parallel to posterior margins of LL, but can be recognised by: S long, with wrinkles, almost parallel to anterior margins of LL (vs. S without wrinkles, extending horizontal in *P.nanyueensis*).

**Male: *Measurements***: Medium-sized. Body length 14.9, DS length 7.7, width 6.6; OS length 6.8, width 4.8. ***Eyes***: AME 0.29, ALE 0.42, PME 0.25, PLE 0.39, AME–AME 0.26, AME–ALE 0.17, PME–PME 0.46, PME–PLE 0.59, AME–PME 0.37, ALE–PLE 0.39, CHAME 0.72, CHALE 0.66. ***Spination***: Pp 131, 101, 2111; Fe I–II 323, III 322, IV 321; Pa I–III 101, IV 000; Ti I–II 2226, III–IV 2126; Mt I–II 2024, III 3025, IV 3036. ***Measurements of palp and legs***: Pp 10.1 (3.3, 1.7, 1.9, –, 3.2), I 33.2 (9.3, 3.0, 9.0, 8.9, 3.0), II 34.8 (9.9, 2.8, 9.5, 9.5, 3.1), III 26.6 (7.7, 2.2, 7.3, 7.0, 2.4), IV 29.5 (8.5, 2.2, 7.7, 8.6, 2.5). Leg formula: II-I-IV-III. Promargin of chelicerae with three teeth, retromargin with four teeth, cheliceral furrow with c. 26 denticles.

***Palp*** (Fig. 4A–C): As in diagnosis. C membranous, arising from T at 11 o’clock position. E expanded and plate-like, arising from T at 9 o’clock position; embolic tip curved. RTA arising basally from Ti; vRTA triangular; dRTA long, with two thin teeth in retrolateral view.

**Figure 6.**
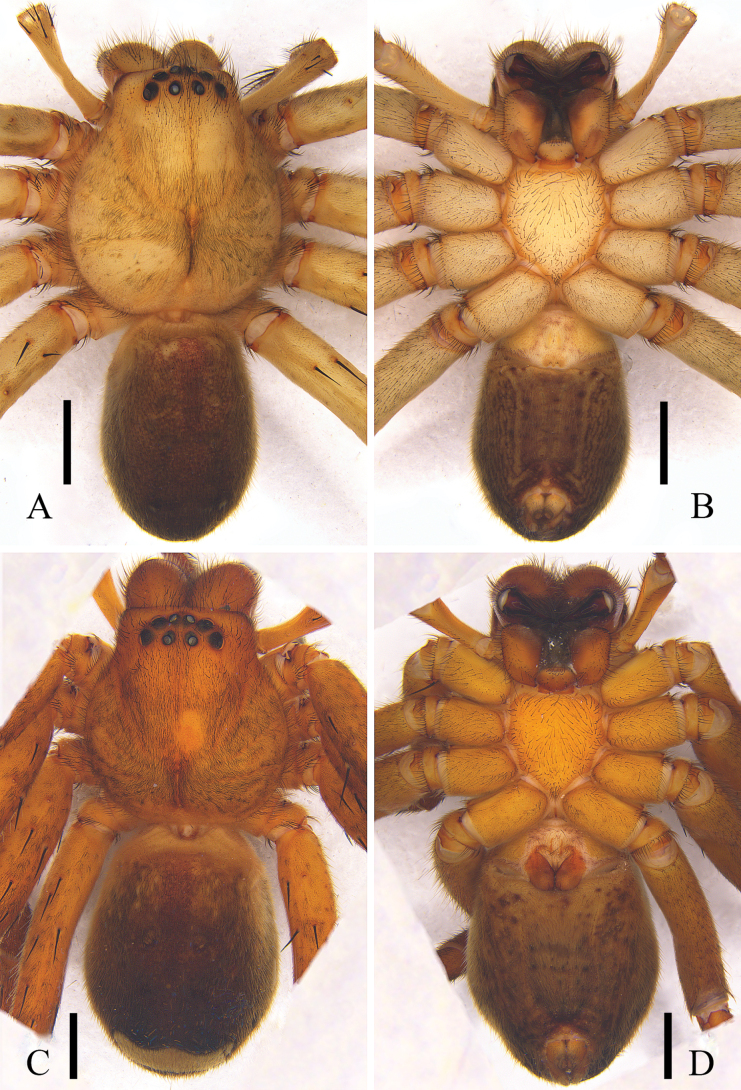
*Pseudopodaqizimeishanensis* Zhang, J. Liu & Hu, sp. nov. **A, B** male habitus (**A** dorsal **B** ventral) **C**, **D** female habitus (**C** dorsal **D** ventral). Scale bars: 2 mm.

***Colouration*** (Fig. 6A, B): DS yellow, with black spots. Ventral legs with black spots. Dorsal OS brown, ventral OS with black spots, spinnerets yellow, with two parallel longitudinal lines of lighter dots.

**Female: *Measurements***: Medium-sized. Body length 16.9, DS length 7.9, width 7.1; OS length 8.6, width 6.6. ***Eyes***: AME 0.33, ALE 0.51, PME 0.31, PLE 0.42, AME–AME 0.32, AME–ALE 0.27, PME–PME 0.58, PME–PLE 0.64, AME–PME 0.44, ALE–PLE 0.41, CHAME 0.60, CHALE 0.58. ***Spination***: Pp 131, 101, 2121, 1014; Fe I–II 323, III 322, IV 321; Pa I–IV 101; Ti I–IV 2026; Mt I 1014, II–III 2024, IV 3025. ***Measurements of palp and legs***: Pp 9.2 (2.8, 1.1, 2.1, –, 3.2), I 25.6 (7.0, 2.9, 7.0, 6.4, 2.3), II 26.9 (7.9, 2.8, 7.2, 6.8, 2.2), III 22.3 (7.0, 2.7, 5.4, 5.2, 2.0), IV 24.6 (7.6, 2.3, 5.9, 6.5, 2.3). Leg formula: II-I-IV-III. Promargin of chelicerae with three teeth, retromargin with four teeth, cheliceral furrow with c. 26 denticles.

***Epigyne*** (Fig. 5A–C): As in diagnosis. EF as wide as long, with obvious AB. Anterior margins of LL V-shaped. S long and anterolaterally pointed, with wrinkles. FW covering entire S. FD narrow.

***Colouration*** (Fig. 6C–D): As in males, but darker and with a transverse white patch in posterior part of dorsal OS.

##### Distribution.

China (Hubei Province) (Fig. 10).

#### ﻿ Pseudopoda weimiani

Taxon classificationAnimaliaAraneaeSparassidae

Zhang, J. Liu & Hu sp. nov.

775C4DE8-700A-57D4-8A85-B6D7C75D1D35

https://zoobank.org/F77D7DED-DBF0-4CB9-907B-051ABD35FD04

Figs 7 , 8 , 9 , 10

##### Type material.

***Holotype*** • male: China, Hubei Province: Enshi Tujia and Miao Autonomous Prefecture, Xuan’en County, Qizimeishan National Nature Reserve, Changtanhe Dong Autonomous Town Qizimeishan Mountain; 30°1'45.19"N, 109°43'45.42"E; elev. 1270 m; 6–11 July 2023; Changhao Hu & Mian Wei leg. (CBEE, QZMS00732). ***Paratypes*** • 2 males and 11 females, with same data as for holotype (CBEE, QZMS02459–QZMS02466, QZMS02779, QZMS04305–QZMS04308) • 1 male, Enshi Tujia and Miao Autonomous Prefecture, Xuan’en County, Qizimeishan National Nature Reserve, Changtanhe Dong Autonomous Town, Liangxihe Village; 29°58'46.33"N, 109°42'11.27"E; elev. 827 m; 29 June 2023; Changhao Hu & Mian Wei leg. (CBEE, QZMS00632) • 1 female, Enshi Tujia and Miao Autonomous Prefecture, Xuan’en County, Qizimeishan National Nature Reserve, Shadaogou Town, Longtan Village; 29°41'42.96"N, 109°39'46.03"E; elev. 637 m; 17 July 2023; Changhao Hu & Mian Wei leg. (CBEE, QZMS00622) • 1 female, Enshi Tujia and Miao Autonomous Prefecture, Xuan’en County, Qizimeishan National Nature Reserve, Shadaogou Town, Baishuihe Village; 29°55'25.78"N, 109°44'9.49"E; elev. 843 m; 23–24 July 2023; Changhao Hu & Mian Wei leg. (CBEE, QZMS03877).

##### Etymology.

This species is named after one of the collectors: Mian Wei; noun in genitive case.

##### Diagnosis.

**Figure 7.**
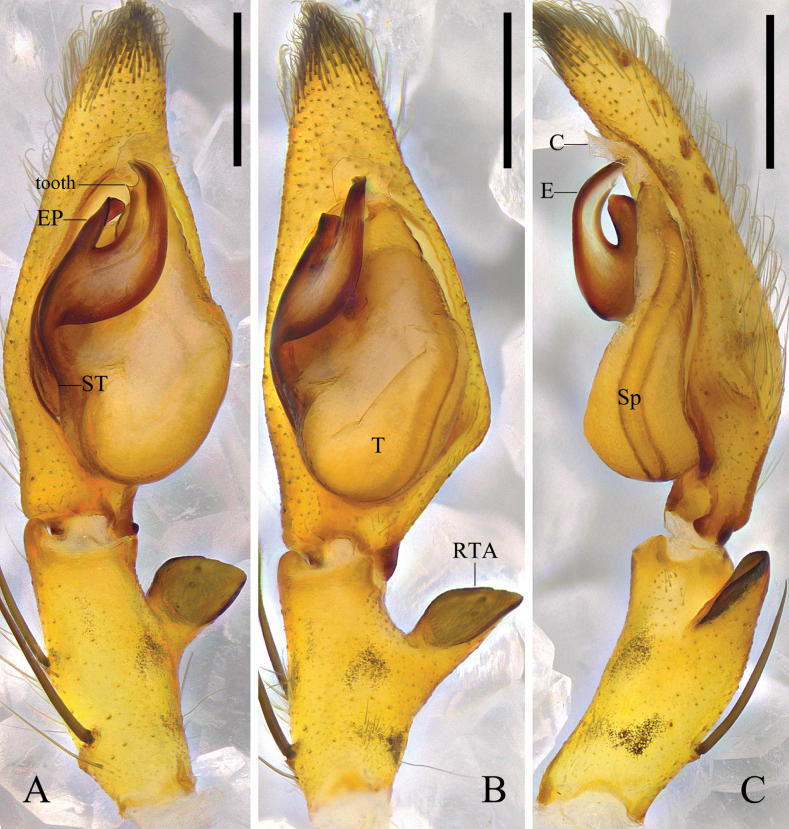
*Pseudopodaweimiani* Zhang, J. Liu & Hu, sp. nov., left male palp (**A** prolateral **B** ventral **C** retrolateral). Abbreviations: C, conductor; E, embolus; EP, embolic projection; RTA, retrolateral tibial apophysis; Sp, spermophor; ST, subtegulum; T, tegulum. Scale bars: 0.5 mm.

**Figure 8.**
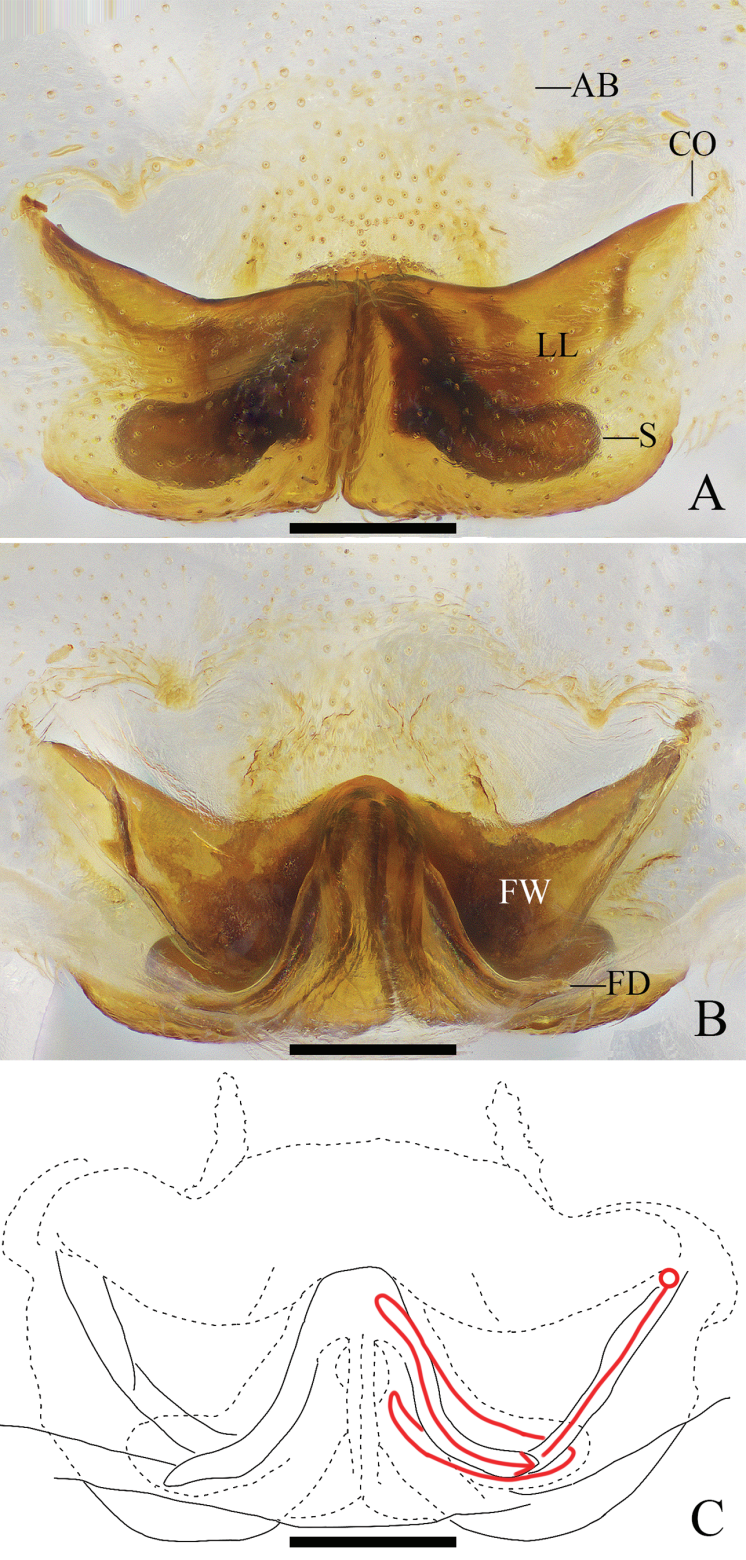
*Pseudopodaweimiani* Zhang, J. Liu & Hu, sp. nov., female **A** epigyne, ventral **B** vulva, dorsal **C** vulva, dorsal; red line represents schematic course of internal duct system. Abbreviations: AB, anterior bands; CO, copulatory opening; FD, fertilization duct; FW, first winding; LL, lateral lobes; S, spermathecae. Scale bars: 0.2 mm.

The male of *P.weimiani* Zhang, J. Liu & Hu, sp. nov. resembles that of *P.hongqi* Deng, Zhong, Irfan & Wang, 2023 (cf. fig. 7A–C vs. figs 5–10 in Deng et al. 2023) by: 1) RTA unbranched; 2) E wide; and 3) EP distinct, but can be recognised by: 1) RTA short and smooth; and 2) EP twisted (vs. RTA long and gradually narrowing toward the tip, EP without a twist, terminal not exceeding E in *P.hongqi*). The female of *P.weimiani* Zhang, J. Liu & Hu, sp. nov. resembles that of *P.taipingensis* Zhang, Jäger & Liu, 2023 (cf. fig. 8A–C vs. fig. 237A–C in Zhang et al. 2023) by: 1) anterior margins of LL almost straight; and 2) S “八”-shaped, but can be recognised by: 1) posterior margins of LL almost straight and parallel to anterior margins; and 2) S with an obvious turning in ventral view (vs. posterior margins of LL W-shaped, S with distinct tube-like structures in *P.taipingensis*).

**Male: *Measurements***: Small-sized. Body length 7.1, DS length 3.6, width 3.2; OS length 3.3, width 2.1. ***Eyes***: AME 0.15, ALE 0.24, PME 0.17, PLE 0.26, AME–AME 0.13, AME–ALE 0.07, PME–PME 0.22, PME–PLE 0.28, AME–PME 0.24, ALE–PLE 0.22, CHAME 0.34, CHALE 0.32. ***Spination***: Pp 131, 101, 2111; Fe I–II 323, III–IV 322; Pa I–III 101, IV 100; Ti I–II 2228, III–IV 2126; Mt I–II 2024, III 3025, IV 3036. ***Measurements of palp and legs***: Pp 5.1 (1.7, 0.6, 1.1, –, 1.7), I 18.8 (4.9, 1.4, 5.9, 4.8, 1.8), II 19.8 (5.5, 1.5, 6.2, 5.1, 1.5), III 14.6 (4.3, 1.0, 4.4, 3.6, 1.3), IV 17.5 (5.0, 1.1, 4.8, 4.8, 1.8). Leg formula: II-I-IV-III. Promargin of chelicerae with three teeth, retromargin with four teeth, cheliceral furrow with c. 26 denticles.

***Palp*** (Fig. 7A–C): As in diagnosis. C membranous, arising from 12 o’clock position of T. E wide, arising from 9 o’clock position of T; EP plate-like and twisted; embolic tip with a small sharp tooth. RTA short, with smooth margin, arising medially from Ti.

**Figure 9.**
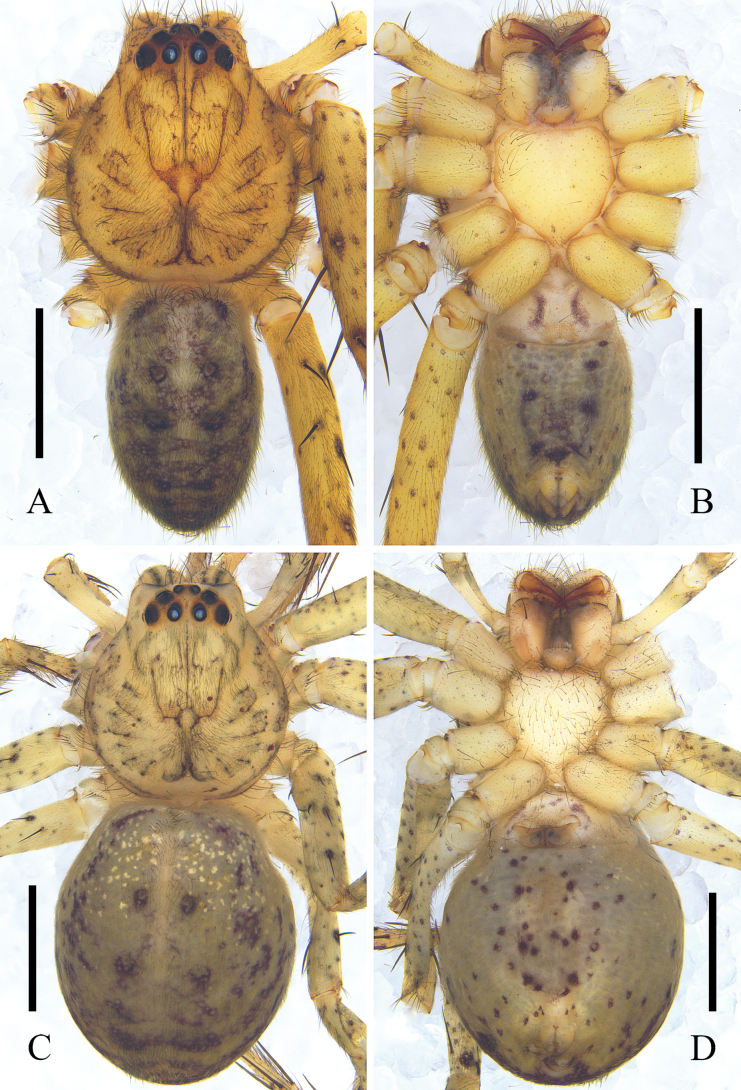
*Pseudopodaweimiani* Zhang, J. Liu & Hu, sp. nov. **A, B** male habitus (**A** dorsal **B** ventral) **C, D** female habitus (**C** dorsal **D** ventral). Scale bars: 2 mm.

**Figure 10.**
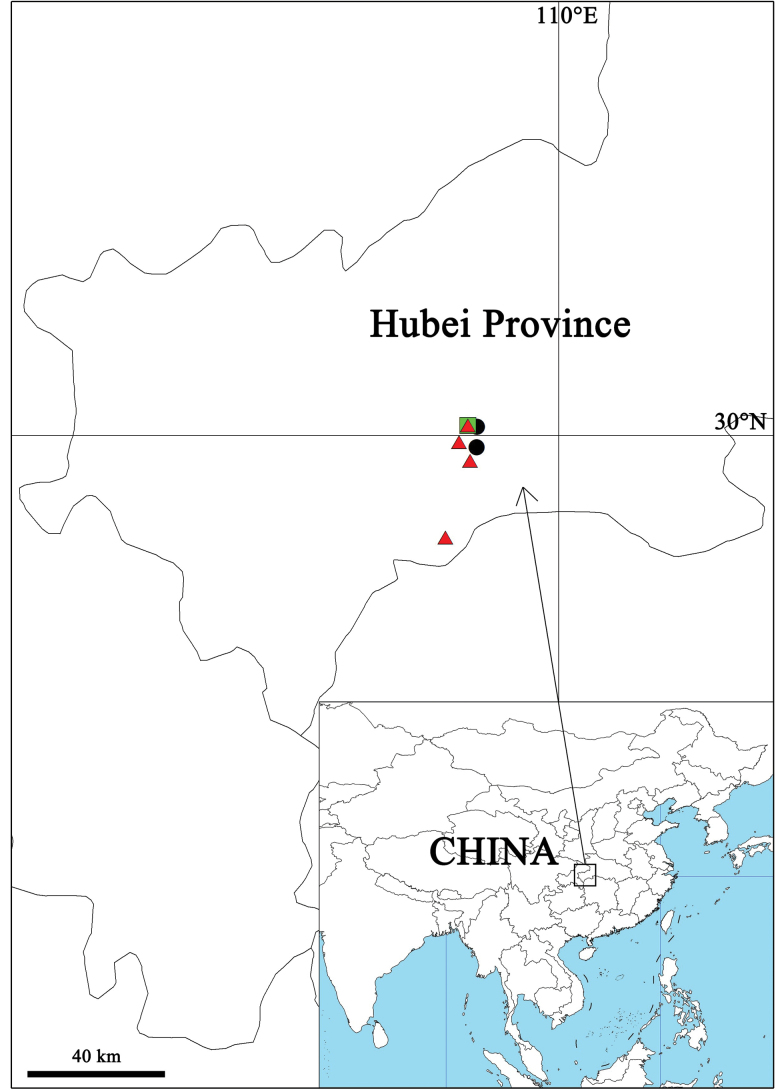
Distribution map of the three new species of *Pseudopoda*. Black circles: *P.arcuata* Zhang, J. Liu & Hu, sp. nov.; green square: *P.qizimeishanensis* Zhang, J. Liu & Hu, sp. nov.; red triangles: *P.weimiani* Zhang, J. Liu & Hu, sp. nov.

***Colouration*** (Fig. 9A, B): DS yellow, with black marked and black margin. Fovea black. Legs with black spots. OS light brown, with several reddish-brown marks, regularly arranged.

**Female: *Measurements***: Small-sized. Body length 8.2, DS length 3.6, width 3.4; OS length 4.5, width 3.9. ***Eyes***: AME 0.15, ALE 0.18, PME 0.23, PLE 0.25, AME–AME 0.15, AME–ALE 0.11, PME–PME 0.22, PME–PLE 0.31, AME–PME 0.25, ALE–PLE 0.30, CHAME 0.32, CHALE 0.26. ***Spination***: Pp 131, 101, 2121, 1014; Fe I–II 323, III–IV 322; Pa I–III 101, IV 100; Ti I–II 2228, IV 2126; Mt I–II 2024, III 3025, IV 3036. ***Measurements of palp and legs***: Pp 4.8 (1.5, 0.7, 1.0, –, 1.6), I 12.0 (3.2, 1.2, 3.4, 3.0, 1.2), II 13.9 (3.9, 1.0, 4.1, 3.6, 1.3), III 10.5 (3.2, 0.9, 2.8, 2.5, 1.1), IV 12.3 (3.8, 0.9, 3.1, 3.3, 1.2). Leg formula: II-IV-I-III. Promargin of chelicerae with three teeth, retromargin with four teeth, cheliceral furrow with c. 26 denticles.

***Epigyne*** (Fig. 8A–C): As in diagnosis. EF wider than long, with indistinct AB. LL much wider than long, anterior margins of LL almost straight, posterior margins almost parallel to anterior margins of LL. Terminal of S twisted, posterolaterally pointed. FW covering anterior part of S. FD long and narrow.

***Colouration*** (Fig. 9C, D): As in males, but with some bright dots in anterior part of dorsal OS.

##### Distribution.

China (Hubei Province) (Fig. 10).

## ﻿Discussion

The genus *Pseudopoda* is widely distributed across China, with 155 species accounting for approximately 61% of the world’s total. The majority of these species are found in the southwestern regions, particularly on the Yunnan-Guizhou Plateau, which harbors 84 species – more than 54% of the Chinese species – highlighting the region’s rich biodiversity and varied topography (Jäger 2001; Jäger and Vedel 2007; Yang et al. 2009; Zhang et al. 2013, 2017; Jiang et al. 2018; Zhang et al. 2019; Yang et al. 2022; Zhang et al. 2023). The distribution of *Pseudopoda* spiders in China is closely linked to the country’s mountainous areas, where species have adapted to specific altitudinal niches. The Qizimeishan National Nature Reserve, part of the northeastern extension of the Yunnan-Guizhou Plateau, features a diverse landscape with elevations ranging from 650 m to over 2010 m (Liu et al. 2006). This variation in altitude creates a variety of microclimates and habitats that support a wide array of species. However, research on the spiders of this area remains limited.

Furthermore, *Pseudopoda* is particularly diverse in high-altitude regions (Jäger, 2001, 2015; Zhang et al. 2023), with approximately 79% of the world’s species found at elevations above 1000 m. In the current study, we report three new *Pseudopoda* species, all of which were collected at elevations above 1000 m, except for *P.weimiani* Zhang, J. Liu & Hu, sp. nov., which was also found at elevations between 600 and 900 m. Finally, the limited dispersal ability of these spiders, likely due to the absence of ballooning behavior, results in small, localized populations, making these species highly susceptible to habitat changes (Bell et al. 2005; Zhang et al. 2021). Consequently, conservation efforts and taxonomic studies should prioritize the Qizimeishan National Nature Reserve to protect its unique and valuable spider fauna.

## Supplementary Material

XML Treatment for Pseudopoda

XML Treatment for Pseudopoda arcuata

XML Treatment for Pseudopoda qizimeishanensis

XML Treatment for Pseudopoda weimiani

## ﻿Additional information

### Author ORCIDs

Jian Chang https://orcid.org/0009-0001-9840-7348

He Zhang https://orcid.org/0000-0002-1478-9837

Jie Liu https://orcid.org/0000-0001-7744-9744

Yang Zhu https://orcid.org/0009-0002-2934-6809

Changhao Hu https://orcid.org/0009-0007-5591-3121

### Data availability

All of the data that support the findings of this study are available in the main text.
